# A time-reversed model selection approach to time series forecasting

**DOI:** 10.1038/s41598-022-15120-x

**Published:** 2022-06-28

**Authors:** Max Sibeijn, Sérgio Pequito

**Affiliations:** Delft Center for Systems and Control, Delft University of Technology, Delft, The Netherlands

**Keywords:** Electrical and electronic engineering, Mechanical engineering, Computational science, Information technology

## Abstract

In this paper, we introduce a novel model selection approach to time series forecasting. For linear stationary processes, such as AR processes, the direction of time is independent of the model parameters. By combining theoretical principles of time-reversibility in time series with conventional modeling approaches such as information criteria, we construct a criterion that employs the backwards prediction (backcast) as a proxy for the forecast. Hereby, we aim to adopt a theoretically grounded, data-driven approach to model selection. The novel criterion is named the *backwards validated information criterion (BVIC)*. The BVIC identifies suitable models by trading off a measure of goodness-of-fit and a models ability to predict backwards. We test the performance of the BVIC by conducting experiments on synthetic and real data. In each experiment, the BVIC is examined in contrast to conventionally employed criteria. Our experimental results suggest that the BVIC has comparable performance as conventional information criteria. Specifically, in most of the experiments performed, we did not find statistically significant differences between the forecast error of the BVIC under certain parameterizations and that of the different information criteria. Nonetheless, it is worth emphasizing that the BVIC guarantees are established by design where the model order penalization term depends on strong mathematical properties of time-reversible time series forecasting properties and a finite data assessment. In particular, the penalization term is replaced by a weighted trade-off between functional dimensions pertaining to forecasting.That said, we observed that the BVIC recovered more accurately the real order of the underlying process than the other criteria, which rely on a static penalization of the model order. Lastly, leveraging the latter property we perform the assessment of the order model (or, memory) of time series pertaining to epileptic seizures recorded using electrocorticographic data. Our results provide converging evidence that the order of the model increases during the epileptic events.

## Introduction

Time series describe a multitude of real-life processes that evolve over time. For instance, consider yearly economic growth, the number of new COVID-19 cases each day, or the electric potential fields measured by an electroencephalographic (EEG) recording. Often, in time-series analysis we seek to determine the underlying rule (or model) that is capable of describing the process (i.e., dependence between adjacent observations). Among the different goals, we often want to forecast how the process is going to evolve within a specified horizon (i.e., up to *h*-steps ahead). Due to uncertainty, time series are generally considered as the realization of stochastic processes often modeled as autoregressive-moving average (ARMA) models, which can be casted as higher order autoregressive models

That said, time series analysis often involves the combination of information criteria and the use of regression techniques to obtain the model parameters. However, regression analysis fails to account for bias due to limited available data^1^. In other words, regression methods are often unable to generalize to data outside the training set. Often, in order to determine the model parameters, one first determines the order of a model (*model selection*), and only then the parameters are estimated (*parameter estimation*). As such, to counter the issue of inherent bias, model selection methods usually contain an order penalization term to avoid over-fitting, and hopefully increase the generalization capabilities. Popular methods for model selection include AIC^2^, and BIC^3^, where the complexity of the model is an indicator for the bias, but also cross-validation, where models are evaluated on their generalization capabilities. In fact, it is also possible to generalize this even further to leave-multiple-out^4^, yet in practice the improvement of performance often does not justify its use. Parameter estimation is often conducted using one of the following methods: Yule–Walker estimation, Burg’s algorithm, maximum likelihood, or least-squares estimation^5^.

An implicit assumption to all previously discussed techniques is that of stationarity (i.e., a time series is said to be stationary if it has similar statistical properties to its time-shifted series)^5^. Real-life processes are almost never stationary in the strict sense, but weaker forms of stationarity exist (e.g. wide-sense stationarity), and may be used to analyse time series that satisfy this assumption. In some cases, such as with an (intracranial) electroencephalography recording^6^, the stationarity assumption holds for limited periods of time. Therefore, data for estimation is constrained to the duration of stationarity. As a result, when the number of samples is small, there is a high probability that methods such as AIC over-fit^7^.

In this paper, we focus on autoregressive processes and we present a novel model selection technique that employs time reversibility properties of autoregressive models to validate forecast ability by evaluating the backwards prediction. Considering past data is accessible, the technique aims to minimize prediction error and uncertainty in models for the backward prediction. Theoretically, we show that backward prediction achieves the same objective as forward prediction.

As such, the new method will be referred to as *backwards validated information criterion (BVIC)*. To perform model selection and determine the model parameters, we formulate an optimization problem that explicitly weighs three features: (a) regression, (b) generalization, and (c) uncertainty. The first component, regression, enables us to evaluate the goodness-of-fit of a specified model with respect to the observed data, and can be quantified through maximum likelihood estimation or least-squares regression. Secondly, generalization represents the ability of a model to predict outside the sample of given observed data, and can be seen analogous to a measure of accuracy of prediction. Lastly, the uncertainty feature of the criterion can be considered as a level of precision exhibited by a prediction. That said, we may quantify the features of generalization and uncertainty through the metrics of mean square prediction error and theoretical prediction variance, respectively.

Consequently, to test the novel model selection criterion we conduct thorough Monte Carlo simulations using experiments with both real and synthetic data. Here, the first two experiments will consist in generating synthetic time series via specified autoregressive models with different model orders and sets of parameters. The third experiment assesses the different criteria when considering intracranial electroencephalographic data. In each experiment, we assess the quality of the BVIC, and other conventional information criteria, on the basis of a selection of performance metrics. Additionally, we evaluate the effects of noise on model selection by varying the noise variance throughout the experiments with synthetic data.

## Methods

Consider an autoregressive process of order *p*, or simply AR(*p*), described by^5^:

1 $$\begin{aligned} X_t - \theta _1 X_{t-1} - \ldots - \theta _p X_{t-p} = W_t, \quad W_t \sim WN(0,\sigma ^2), \end{aligned}$$

where $\{X_t \in {\mathbb {R}}: t \in {\mathbb {N}}\}$ is a stochastic process, with parameters $\{\theta _i \in {\mathbb {R}}: i = 1,\dots , p\}$. The process noise is an independent and identically distributed (i.i.d.) Gaussian white noise (WN) sequence, $\{W_t \in {\mathbb {R}}: t \in {\mathbb {N}}\}$, with variance $\sigma ^2 \in {\mathbb {R}}^+$. The linear prediction $X_{n+h}^n$ denotes the *h*-step ahead predictor using the last *n* measurements which is described as^8^

2 $$\begin{aligned} X_{n+h}^n = \theta _{n1}^{(h)} X_n + \theta _{n2}^{(h)} X_{n-1} + \ldots + \theta _{nn}^{(h)} X_{1} = (\theta _n^{(h)})^\intercal X { = f_n^h(X)}, \end{aligned}$$

where $X = (X_n, X_{n-1}, \ldots , X_1)^\intercal \in {\mathbb {R}}^n$ and $\theta _n^{(h)} \in {\mathbb {R}}^n$ for forecast horizon $h \in {\mathbb {N}}$. Note that the predictor is described to at most *n* parameters. In practice, one would consider an estimate of *j* non-zero parameters.

We are interested in finding a model that follows the linear structure in (2) that *best* represents the true process as described in (1), according to some predefined metric. Selection of models is concerned with finding a parametric model for a specified objective, such as minimal error between forecasted and measured data. In order to find the predictor that obtains minimum mean squared error we use Theorem 1—see S1 Supplementary Information.

Ideally, we find the optimal linear predictor by minimizing the mean squared error over a specified prediction horizon as follows:

3 $$\begin{aligned} \min _{f_j} \frac{1}{h_2-h_1+1}\sum _{i=h_1}^{h_2} E[(X_{n+i} - f_j^i(X))^2 \mid X], \quad j \in {\mathscr {M}}, \end{aligned}$$

for some $h_1,h_2 \in {\mathbb {N}}$, and $h_2 > h_1$. Subsequently, it readily follows that (3) is minimized when

4 $$\begin{aligned} { f_j^i(X) = E[X_{n+i} \mid X], \quad \forall i \in [h_1, h_2].} \end{aligned}$$

Notice that it is only possible to determine $f_j^i(X)$ when the probability distribution of $X_{n+i}$ is known, which is not the case. Alternatively, if measured values of $x_{n+i} \in {\mathbb {R}}$ are known, we may use them to construct an empirical distribution. In other words, measured data can be used to fit a model. However, if we consider the objective to find a model for forecasting, future values are not at our disposal. Thus, instead of using future values to obtain an empirical distribution, past (i.e., available) data must be used to find this function. Model selection for forecasting is concerned with finding models that approximate the probability distribution of the underlying process.

### Model selection

In the case of linear processes, specifically an AR(*p*) process considered in (1), model selection can be divided into two components: (1) order selection and (2) parameter estimation.

Order selection and parameter estimation can be performed simultaneously. For a set candidate model orders ${\mathcal {M}} = \{1,\ldots ,m\}$, parameters are estimated for each model order $j \in {\mathcal {M}}$. Subsequently, candidate models are assessed based on a metric that describes the *goodness-of-fit* of a model with respect to the data. Often, the log-likelihood function is used as a measure of goodness-of-fit, where a greater log-likelihood is associated with a better fit^1^. Usually, the maximum likelihood (ML) estimate or the least squares (LS) estimate may be used to approximate the log-likelihood^9^. At the same time, these methods serve as *estimators* for the parameters upon the data^5^.

In practice, when observations are limited, it is difficult to precisely capture the underlying structure of a process. For instance, when we consider the ML estimator is used to estimate the parameters of a model, the measure of goodness-of-fit is biased and the comparison of different models is not fair. Essentially, the bias is caused by reusing the same data for estimation and for evaluation, resulting in a preference of complex models (i.e., with very high values of *p*)—i.e., over-fitting^1^. In other words, models with maximum log-likelihood are a very good fit of the observed data, but tend to display poor predictive ability.

Typically, to address the problem of over-fitting, model selection is conducted by using *information criteria*. Candidate models are assessed as a function of maximum likelihood and an additional penalization (or regularization) term. Often, these information criteria penalize the number of parameters, to prevent over-fitting. For instance, the Akaike information criterion (AIC)^2^ described by

5 $$\begin{aligned} \text {AIC}(j) = -2 \ell ({ {\hat{\theta }}_j}) + 2j, \end{aligned}$$

for an estimated model with parameters ${ {\hat{\theta }}_j}$, with $j\in {\mathcal {M}}$, representing the number of model parameters. For small samples, the AIC still has a high probability of over-fitting^7^. Therefore, the corrected version of AIC (AICc) is often considered in such cases. The AICc is formulated as

6 $$\begin{aligned} \text {AIC}_c(j) = \text {AIC}(j) + \frac{2j(j+1)}{n-j-1}, \end{aligned}$$

where $n \in {\mathbb {N}}$ represents the number of observations. A third method for model selection is the Bayesian information criterion (BIC)^3^ described by

7 $$\begin{aligned} \text {BIC}(j) = -2 \ell ({ {\hat{\theta }}_j}) + j \log n. \end{aligned}$$

That said, while information criteria are commonly used to reduce over-fitting, their properties are only meaningful when the number of samples approach infinity. For instance, AIC is asymptotically efficient^10^, meaning that when $n \rightarrow \infty$, AIC chooses a model $f(X) \rightarrow E[Y \mid X]$. However, for $n < \infty$, AIC offers no guarantees on the ability of a specified model to generalize outside of its sample with respect to any other model.

Let us now introduce a different situation. Consider the process described in (1). Instead of trying to find the optimal linear predictor for the *h*-step ahead prediction, we want to find the optimal linear predictor for the *h*-step back prediction. The predictor can be formulated as^8^

8 $$\begin{aligned} X_{1-h}^n = \theta _{n1}^{(h)} X_1 + \theta _{n2}^{(h)} X_{2} + \ldots + \theta _{nn}^{(h)} X_{n} = (\theta _n^{(h)})^\intercal X_B = f_{n,B}^h(X_B), \end{aligned}$$

where $X_B = (X_1, X_2, \ldots , X_n)^\intercal \in {\mathbb {R}}^n$ is a reversed version of *X*, and $\theta _n^{(h)} \in {\mathbb {R}}^n$ for backcast horizon $h \in {\mathbb {N}}$. We present a similar argument as in (3) to minimize the mean square error for the backwards prediction, as follows:

9 $$\begin{aligned} \begin{aligned} \min _{f_{j,B}} \frac{1}{h_2-h_1+1}\sum _{i=h_1}^{h_2} E[(X_{1-i} - f_{j,B}^i(X_B))^2 \mid X_B], \quad j \in {\mathcal {M}}. \end{aligned} \end{aligned}$$

Consequently, it follows—see S5 Supplementary Information for details—that (3) is minimized when

10 $$\begin{aligned} f_{j,B}^i(X_B) = E[X_{1-i} \mid X_B], \quad i \in [h_1,h_2]. \end{aligned}$$

Again, we can approximate $f_{j,B}^i(X_B)$ if we have an approximation of the distribution of  $X_{1-i}$. However, in contrast to the forward prediction, we can now construct an empirical distribution on the basis of past data. Therefore, we are able to find $f_{j,B}^i(X_B)$ for all $i \in \{h_1,\dots ,h_2\}$.

**Figure 1:**
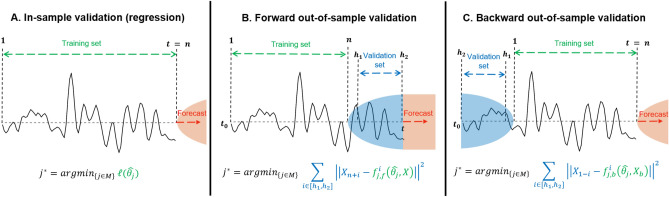
Model validation schemes. Three different possibilities for model validation are illustrated. In (**A**), the training set is used for training and validation. In (**B**), a section from the front of the set is used for validation. In (**C**), a section from the back is used for validation. The set of observations ranges between $t_0$ and *t*. Below each scheme, the associated equation to find the optimal order is given. Note that for illustrative purposes the prediction steps are indicated using the number of steps from the point of prediction (i.e., not the actual value in the set). For B, the actual values correspond to $n+h_1$, and $n+h_2$, while in (**C**, the values correspond to $1-h_1$, and $1-h_2$.

In order to further leverage the argument presented in this section, we present an illustrative example of three separate validation schemes, of which the first is in-sample, and the other two are out-of-sample. The illustration is given in Fig. 1. The three cases are explained as follows according to an arbitrary set of observations, $x_k$, where $k \in [t_0, t]$:

(A) For in-sample model validation (i.e., regression), the same data is used for training as for validation. This method does not allow for out-of-sample validation because no observations $x_k$, for $k > n$, exist. It is advantageous in the sense that more data can be used for training, but has high risk of overfitting. Therefore, it often does not generalize well, making it unsuitable for forecasting.
(B) In the second case, we split the observations such that it is possible to perform a out-of-sample validation scheme using a forecast. This is possible because the set of observations $\{x_k$: $k > n\}$, is non-empty. However, the ensuing model is trained and validated to forecast from time instance *n*, and not *t*. Forecasting more than $h_2$ steps ahead is generally bad practice (depending on $h_2$), as the uncertainty of the forecast will grow too large. Alternatively, one cannot simply assume that the model obtained using only the points $x_k$ for $k = 1,\dots ,n$ is applicable to the entire set of observations (i.e., $x_k, k = t_0,\dots ,t$). In fact, this would make it an in-sample model, and the same argument as in A holds. Therefore, we argue that this approach is not suitable for forecasting.
(C) In the final case, we split the data opposite to B, with the validation set before the training set. Suppose that we temporarily reverse the time axis (i.e., mirror the plot from C). We can present a similar argument as in B, and do out-of-sample validation on the backcast because the set $\{x_k: k < 1\}$ is non-empty. However, in constrast to B, we can now use properties of time-reversibility (to be discussed in the next section) to show that the set of parameters (i.e., the model) is entirely independent of the direction of time. Therefore, we can apply the mirrored model without suffering from a ‘time gap’ between *n* and *t*, resulting in an out-of-sample model that can forecast from the present time, *t*.

In the next section, we argue that, from a theoretical perspective, $f_{j,B}^i(X) = f_j^i(X)$.

### Reversibility of time series

A time series is time-reversible when the sequence of random variables $\{X_t,\dots ,X_{t+h}\}$ has equal joint probability distribution to the sequence $\{X_{t+h},\dots ,X_{t}\}$^11^. Standard ARMA models driven by Gaussian noise are time-reversible^12^. Therefore, ARMA models are independent of the direction in which time progresses. In the remaining of this paper, and without loss of generality, we focus on autoregressive processes (AR).

That said, let us consider a case where we want to compare the one-step ahead prediction with the one-step backward prediction making use of *n* measurements. The linear prediction of the one-step ahead prediction is given by^13^

11 $$\begin{aligned} X_{n+1}^n = \varphi _{n1}X_n + \varphi _{n2}X_{n-1} + \ldots + \varphi _{nn}X_{1} = \varphi _n^\intercal X, \end{aligned}$$

with $\varphi _n \in {\mathbb {R}}^n$. The linear prediction for the one-step backcast is described by

12 $$\begin{aligned} X_0^n = \theta _{n1}X_1 + \theta _{n2}X_2 + \ldots + \theta _{nn}X_n = \theta _n^\intercal X_B, \end{aligned}$$

with $\theta _n \in {\mathbb {R}}^n$. In this case the vector containing the states is $X = (X_n, X_{n-1},\ldots ,X_1)^{\intercal }$. Here, $X_B$ denotes a time reversed vector of *X* which is formulated as $X_B = JX$, where $J \in {\mathbb {R}}^{n\times n}$ is an anti-diagonal identity matrix described as follows:

$$\begin{aligned} J =\left( \begin{array}{cccccc} 0 &{} \quad 0 &{} \quad \ldots &{} \quad 0 &{} \quad 1 \\ 0 &{} \quad 0 &{} \quad \ldots &{} \quad 1 &{} \quad 0 \\ \vdots &{} \quad \vdots &{} \quad &{} \vdots &{} \quad \vdots \\ 0 &{} \quad 1 &{} \quad \ldots &{} \quad 0 &{} \quad 0 \\ 1 &{} \quad 0 &{} \quad \ldots &{} \quad 0 &{} \quad 0 \end{array}\right) . \end{aligned}$$

Next, the Yule–Walker equations (or, *prediction equations*) can be used to determine the parameters for both the predictors as a function of the autocovariance function of the ${X_t}$. The Yule–Walker equations are described as follows^13^:

13 $$\begin{aligned} \begin{aligned} \Gamma _n \varphi _n= & {} \gamma _n, \quad \text {for the forward prediction, and}\\ \Gamma _n \theta _n= & {} \gamma _n, \quad \text {for the backward prediction.} \end{aligned} \end{aligned}$$

Their corresponding variances are given by

14 $$\begin{aligned} \begin{aligned} \sigma _f^2= & {} \gamma (0) - \varphi _n^\intercal \gamma _n, \quad \text {for the forward prediction, and}\\ \sigma _b^2= & {} \gamma (0) - \theta _n^\intercal \gamma _n, \quad \text {for the backward prediction.} \end{aligned} \end{aligned}$$

In these equations, $\Gamma _n \in {\mathbb {R}}^{n\times n}$ is the autocovariance matrix described by

$$\begin{aligned} \Gamma _n = \left( \begin{array}{cccccc} \gamma (0) &{} \gamma (1) &{} \ldots &{} \gamma (n-2) &{} \gamma (n-1) \\ \gamma (1) &{} \gamma (0) &{} \ldots &{} \gamma (n-3) &{} \gamma (n-2) \\ \vdots &{} \vdots &{} \ddots &{} \vdots &{} \vdots \\ \gamma (n-2) &{} \gamma (n-3) &{} \ldots &{} \gamma (0) &{} \gamma (1) \\ \gamma (n-1) &{} \gamma (n-2) &{} \ldots &{} \gamma (1) &{} \gamma (0) \end{array}\right) ,\end{aligned}$$

and $\gamma _n = \{\gamma (i):i=1,\dots ,n\}$ is the vector of autocovariances up to *n* lags. Both $\Gamma _n$ and $\gamma _n$ consist of values from the autocovariance function of $\{X_t\}$, which is symmetric. This means that $\gamma _X(k) = \gamma _{X_r}(k)$. Therefore, the function is independent of the direction of time in which the time series is ordered. Hence, we have that $\varphi _n = \theta _n$.

#### *Example*

To further illustrate the property of time-reversibility, consider an AR(1) process described by

15 $$\begin{aligned} X_t = \alpha X_{t-1} + W_t, \quad W_t \sim WN(0,1), \quad t = 0, 1, \dots \end{aligned}$$

If the Yule–Walker equations are applied for the forward prediction as described in (11), we obtain

16 $$\begin{aligned} \begin{aligned} \gamma (0) \varphi _{11} = \gamma (1). \end{aligned} \end{aligned}$$

Alternatively, the autocovariance $\gamma (k)$ is found by taking the expectation of the process from (15) with a shifted version of itself as follows:

17 $$\begin{aligned} \begin{aligned} \gamma (k)&\; = E[X_tX_{t-k}]\\&{\mathop {=}\limits ^{(15)}} \alpha E[X_{t-1}X_{t-k}] + \underbrace{E[{ W_t} X_{t-k}]}_{=0} \\&\; = \alpha \gamma (k-1). \end{aligned} \end{aligned}$$

Now, using the result from (16) and (17), and the fact that the forward and backward parameter vectors are the same, we can conclude that

18 $$\begin{aligned} \begin{aligned} \varphi _{11}&= \theta _{11} = \alpha , \; \text {and}\\ \sigma _f^2&= \sigma _b^2 = \gamma (0) - \alpha \gamma (1). \end{aligned} \end{aligned}$$

This results suggests that the model for the one-step ahead prediction is identical to the model of the one-step backward prediction.

In summary, the above derivation is readily applicable to all orders of an AR(*p*) model, and also for different prediction horizons. Thus, this means that $f_{j,B}^i(X) = f_j^i(X)$. As such, we can leverage previous data to assess the quality of the backcasting, which serves as a proxy to the forecasting capabilities. In the next section, we systematically leverage this insight to introduce a novel information criteria that builds upon these ideas.

## Backwards validated information criterion

We propose a novel information criterion called the *backwards validated information criterion (BVIC)*—not to be confused with BIC (i.e., Bayesian information criterion). The BVIC is designed to estimate the order of of an AR(*p*) process. Therefore, we consider a range of candidate autoregressive model orders up to a specified maximum order $m \in {\mathbb {N}}$ , where a single candidate model order is defined as $j \in {\mathcal {M}}$, with ${\mathcal {M}} = \{1,2,\dots ,m\}$. The BVIC is given by

19 $$\begin{aligned} \mathrm {BVIC}(j) = -\frac{\ell ({{\hat{\theta }}_j})}{|\ell ({\hat{\theta }}_{m})|} + \beta \frac{\mathrm {err}({{\hat{\theta }}_j^{\tiny {YW}}})}{\mathrm {err}({{\hat{\theta }}}_{m}{\tiny {YW}})} + \gamma \frac{ \mathrm {var}({{\hat{\theta }}_j^{\tiny {YW}}})}{\mathrm {var}({{\hat{\theta }}}_{m}^{\tiny {YW}})}, \end{aligned}$$

where $\beta , \gamma \ge 0$. The set of parameters for a specified model $j \in {\mathcal {M}}$ is described by either ${\hat{\theta }}_j \in {\mathbb {R}}^j$, ${\hat{\theta }}_{m} \in {\mathbb {R}}^m$, denoting the parameters obtained through ML estimation, or by ${\hat{\theta }}_j^{{{YW}}} \in {\mathbb {R}}^j$, ${\hat{\theta }}_{m}^{{{YW}}} \in {\mathbb {R}}^m$, denoting the parameters obtained through Yule–Walker estimation. The denominators of the BVIC, depending on *m*, act as a normalization of the criterion, this ensures that the components are weighted more equally and makes the choice for $\beta$ and *gamma* more intuitive. Moreover, the criterion contains three functions that depend on ${\hat{\theta }}_j$: ($i$) the *log-likelihood* function denoted by $\ell (\cdot )$, ($ii$) the *mean square backcast error* over horizon interval $[h_1,h_2]$, denoted by err($\cdot$), and ($iii$) the *mean variance* of backcasted values, denoted by var($\cdot$)—see S1 Supplementary Information for the details in the derivation of the BVIC. That said, we emphasize that the BVIC is to be used only as an index criterion. In other words, the BVIC is used to give a quantitative assessment of each model order (i.e., to perform model identification), and it does not play a role in parameter estimation. Parameters are estimated using the Yule–Walker equations with the order selected by the BVIC.

Consider a set of observations described by $X = \{X_{t-h} : t = 1,\ldots , n\} \in {\mathbb {R}}^n$, with number of samples $n \in {\mathbb {N}}$, and backcasting horizon interval $h_1,h_2 \in {\mathbb {N}}$. The *h*-step linear backwards prediction can be denoted as $X_{1-h}^j = \theta _j^\intercal X_{1:j}$, with $\theta _j, X_{1:j} \in {\mathbb {R}}^j$. Consequently, we can write each of the discussed components of the BVIC as a function of the respective estimated parameters, as follows:

20 $$\begin{aligned} \begin{aligned} \ell ({ {\hat{\theta }}_j }) =&-\frac{n-p}{2} \log {{\hat{\sigma }}({ {\hat{\theta }}_j })^2},\\ \mathrm {err}({ {\hat{\theta }}_j^{{YW}} }) =&\frac{1}{h_2-h_1+1}\sum _{i=h_1}^{h_2} (X_{1-i} - X_{1-i}^j({ {\hat{\theta }}_j^{{YW}} }))^2, \text { and}\\ \mathrm {var}({ {\hat{\theta }}_j^{{{YW}}} }) =&\frac{1}{h_2-h_1+1}\sum _{i=h_1}^{h_2}{P_{1-i}^j({ {\hat{\theta }}_j^{{YW}} })}, \end{aligned} \end{aligned}$$

where $P_{1-i}^j({\hat{\theta }}_j^{ {{YW}}}) = E[(X_{1-i} - X_{1-i}^j({\hat{\theta }}_j^{ {{YW}}}))^2] = \sigma _X^2(1 - ({\hat{\theta }}_j^{ {{YW}}})^\intercal \rho _n(i))$, with $\sigma _X^2 \in {\mathbb {R}}^+$ being the variance of *X* and $\rho _n(i) = (\rho (i),\ldots ,\rho (i+p)) \in {\mathbb {R}}^p$ is a vector of autocorrelations.

**Figure 2:**
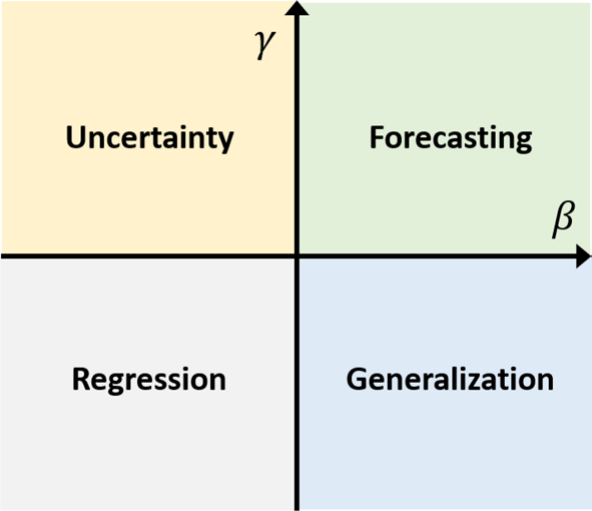
Dimensions of the BVIC. The four dimensions of the BVIC divided into four quadrants depending on the size of parameters $\beta$ and $\gamma$. The intersection of the arrows does *not* represent the point at which $\beta ,\gamma = 0$.

Interestingly, the BVIC can be divided into four dimensions: (*a*) regression, (*b*) generalization, (*c*) uncertainty and (*d*) forecasting. Each dimension illustrates a functionality of the criterion with regards to time series analysis. The four dimensions are depicted in four quadrants in Fig. 2. Each quadrant contains the functionality of the BVIC depending on the selection of the parameters $\beta$ and $\gamma$. For instance, if $\beta ,\gamma = 0$, the BVIC is equal to the ML estimate. When $\gamma = 0$ and $\beta \gg 1$, the BVIC selects models with the smallest out-of-sample error (on the backcast) that intuitively corresponds to generalization. When both $\beta , \gamma \gg 1$, the focus of the BVIC is to minimize the out-of-sample error along with the theoretical variance (uncertainty) of the backcast, thereby also minimizing these quantities for the forecast.

That said, recall that a forecast consists of both the point and variance estimate, and as such, they both play a key role in forecasting that simply speaking would relate with the precision and accuracy of the predictions. Thus, the different dimensions associated with the parameters of the BVIC give a principle approach to model selection, contrasting with the classical information criteria previously discussed (i.e., AIC, AICc, and BIC). Specifically, this is achieved by replacing the penalization of the order with a regularization term on the backward validation metric and adding parameters that introduce adaptability in the the model selection problem. In other words, the adaptability allows users of the BVIC to select their preferred forecasting goal.

Moreover, one must also select a forecast horizon interval $h_1,h_2$ when using the BVIC. In general, the selection of a look-ahead interval (not necessarily consecutive) depend on the application. For instance, if you want to use the BVIC to validate a model, you can use the one-step ahead prediction. On the other hand, if you consider applications where you want to forecast further into the future, such as prediction of epileptic seizures, then you may consider a larger forecast horizon, or even a concentrated range of steps in the future.

## Simulations

In what follows, we will perform Monte Carlo simulations using three experiments with both synthetic and real data. Specifically, the first and second experiments will consist in generating data according to specified autoregressive models, and assess the model order obtained through different information criteria, as well as the goodness-of-fit. Next, we assess the quality of the results when considering intracranial electroencephalographic (i.e., electrocorticographic, or ECoG for short) data from epileptic patients undergoing a seizure.

### Data description

Firstly, for the synthetic data we generate an autoregressive process of order $p \in {\mathbb {N}}$ and parameters $\theta \in {\mathbb {R}}^p$ as described in (1). Subsequently, similar to^6^, we add noise to the synthetic data. First, the realization is standardized such that the mean and variance are equal to zero and one, respectively. Second, the measurement noise is added as follows:

21 $$\begin{aligned} Y_t = X_t + \delta Z_t, \quad \{Z_t\} \sim {\mathcal {N}}(0,1), \end{aligned}$$

from which we obtain ${\mathbb {Y}} = \{Y_t : t = 1,2,\dots , N\} \in {\mathbb {R}}^N$ that contains $N \in {\mathbb {N}}$ measurements. Note that *N* is used to describe the entire generated sample size, whereas *n* denotes the effective sample size that may be used for training (and validation). Moreover, the sequence $\{Z_t\}$ is i.i.d., and the parameter $\delta \ge 0$ may be used to determine the signal-to-noise ratio (SNR).

Secondly, the ECoG data is obtained from the International Epilepsy Electrophysiology Portal (IEEG Portal)^14^. We look at a range of channels from three different patients from two different locations. The first and second datasets are acquired from two separate patient studies at the Hospital of the University of Pennsylvania, Philadelphia, where the ECoG signals were recorded at a sampling frequency of 512 Hz. The third dataset is recorded at a frequency of 500 Hz, and is from a patient study at the the Mayo Clinic in Rochester, Minnesota.

Seizures are marked by clinical experts^15^ and the seizure-onset time and location are defined by the so called *earliest electrographic change (EEC)* and the *unequivocal electrographic onset (UEO)*^16^, where we consider the period between EEC and UEO to be the pre-ictal phase, i.e., the phase between a normal (interictal) state and a seizing (ictal) state.

We extract univariate time series blocks from channels in which seizures were identified. Each block has been associated to one of three states of the brain, being: ($i$) interictal, ($ii$) pre-ictal, or ($iii$) ictal. Subsequently, two steps of pre-processing were performed on the data. Initially, the common reference was removed from all the recorded data. Hereafter, each recording is filtered through a 60 Hz notch filter to remove line-noise present in the recordings. Both these steps were also performed in^15^, where the same database is used.

### Experimental setup

A specific realization of *Y* is referred to as a *window* (of data collected over a period of time). A single window is denoted by $w_q \in {\mathbb {R}}^{N}$, with $q = 1,2,\dots ,{ n}_w$. In each experiment, we generate a collection of ${ n}_{w} \in {\mathbb {N}}$ windows.

**Figure 3:**
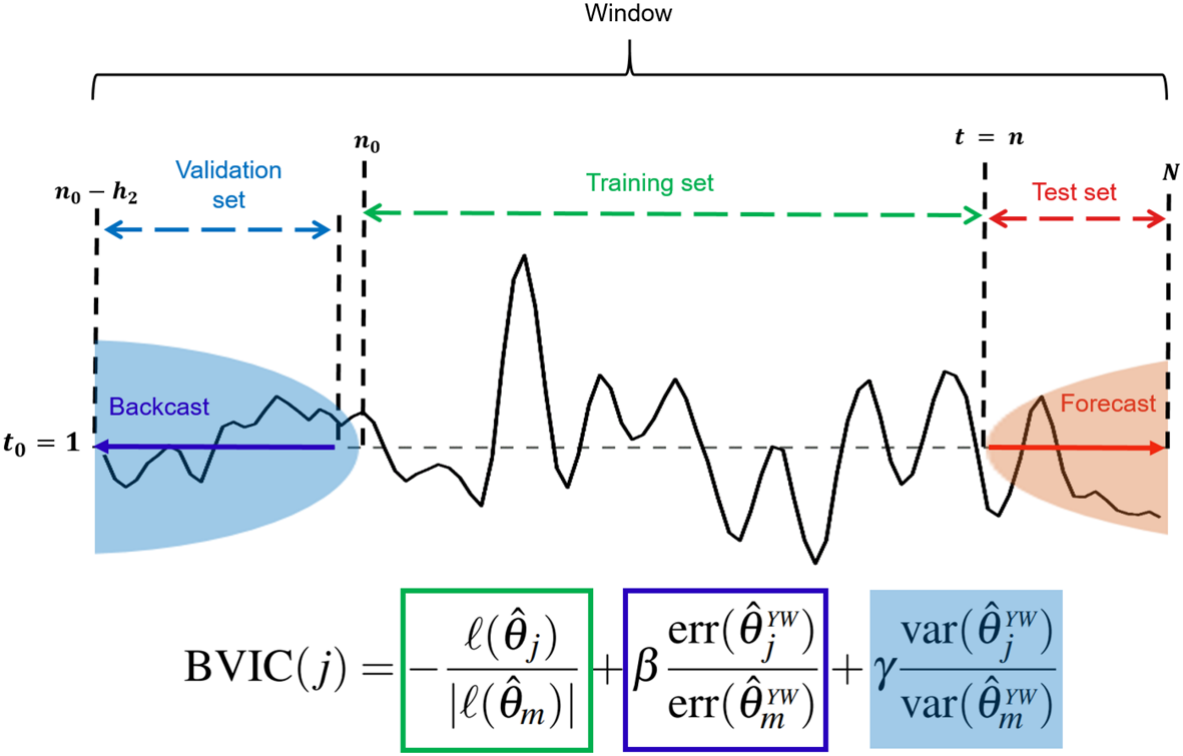
Data splitting. This figure depicts how each data segment (i.e., window) is divided into training and validation set. Special emphasis is drawn to the backward validation scheme required to assess the performance of the BVIC.

Each window is split into three parts. Firstly, a training set ${\mathcal {T}}_q := \{w_{q,t} : t = n_0,\dots , n\}$. Secondly, a validation set ${\mathcal {V}}_q := \{w_{q,t}:t=1,\dots , n_0-1\}$. Thirdly, a test set ${\mathcal {T}}^*_q := \{w_{q,t}:t=n+1,\dots , N\}$. See Fig. 3 for an illustration of the split. For the conventional criteria that use in-sample validation, the training and validation set are combined.

We set $h_1 = 1$ to test the BVIC over the entire horizon. This means that ${\mathcal {V}}_q,{\mathcal {T}}^*_q \in {\mathbb {R}}^{h_2}$. The size of the combined training and validation set corresponds to *n*.

The respective sizes of the training and validation set depend on the true order (which is known) of the autoregressive process and the forecasting horizon $h_2 \in {\mathbb {N}}$. Specifically, we choose $n = 2(p + h_2)$ and $S_{{\mathcal {T}}^*} = h_2$. As a result, when $p = h_2$, we have training and testing split of $n= 4h_2$ (80%) and $|{\mathcal {T}}^*_q| = h_2$ (20%). Subsequently, to ensure that the windows have sufficient samples, the window size is chosen to be $N = S_{{\mathcal {T}}} + 2S_{{\mathcal {T}}^*}$. Finally, the windows are always normalized (z-scored) to facilitate a fair evaluation.

### Metrics

The results from the Monte Carlo simulations are evaluated based on three metrics. The first two metrics are based on the $\ell _2$-loss function of the forecast (i.e., the mean squared error over the forecast horizon). This metric is computed as follows:

22 $$\begin{aligned} L_{w, m}^2(h_2) = \frac{1}{h_2}\sum _{i=n+1}^{n+h} (Y_{i,q,m} - Y_{i,q,m}^j)^2, \end{aligned}$$

where $q \in \{1, 2, \ldots , n_w\}$ is the index of the window, and $m \in \{1, 2, \ldots , n_m\}$ is the index of the Monte Carlo simulation. The metrics MSE and VAR are calculated by respectively taking the mean and variance over all the windows and simulations as follows:

23 $$\begin{aligned} \begin{aligned} \text {MSE}&= \frac{1}{n_w n_m}\sum _{m=1}^{n_m} \sum _{q=1}^{n_w} L_{q,m}^2(h_2), \quad \text {and}\\ \text {VAR}&= \frac{1}{n_w n_m}\sum _{m=1}^{n_m} \sum _{q=1}^{n_w} (L_{q,m}^2(h_2) - \text {MSE})^2. \end{aligned} \end{aligned}$$

Additionally, we include prediction uncertainty of the forecast as an evaluation metric by taking the average variance over the forecast horizon, i.e.,

24 $$\begin{aligned} P_{q,m} = \gamma _{q,m}(0) - \frac{1}{h}\sum _{i=1}^{h}\gamma _{j,q,m}^{'(i)} \Gamma _{j,q,m}^{-1} \gamma _{j,q,m}^{(i)}. \end{aligned}$$

Subsequently, the mean over all simulations can be computed as

25 $$\begin{aligned} \bar{P}_F = \frac{1}{n_m n_w}\sum _{m=1}^{n_m} \sum _{q=1}^{n_w} P_{q,m}. \end{aligned}$$

### Experiments

#### Experiment 1

In this experiment, we test the ability of the different models determined using the different information criteria to forecast different time series. We evaluate four different synthetic AR(5) models by conducting Monte Carlo simulations for the BVIC and benchmark criteria. Since the autoregressive model is a discrete linear filter, the parameters of the model can be determined when the poles (or, roots) of the system are known^13^. For the autoregressive process to be stationary, the poles of the system need to lie inside the unit circle. The location of the poles affect the frequency behaviour and exponential decay of the time domain signal. For instance, a larger phase angle of a complex conjugate pole set results in higher frequency of the time domain signal. Essentially, the dominant pole(s) (i.e., the poles that lie nearest to the unit circle) of the system determine the majority of this behaviour. Therefore, we define four sets of dominant poles that display different response behaviour to assess the different information criteria.

**Case 1.**: This set of poles chosen to be similar to the poles of a true ECoG recording. These poles are computed using a least squares system identification method detailed in S2 Supplementary Information. The dominant poles are a positive real pole of $z = 0.9$, and set of complex conjugate poles with positive real part, $z = 0.6 \pm 0.6i$. This results in a frequency of $\omega _0 = 0.79$ rad/s. The magnitude of the poles will lead to slow exponential decay while the phase angle of the complex conjugate will induce intermediate sinusoidal behaviour.
**Case 2.**: The dominant poles are a set of complex conjugate poles with negative real part, $z = -0.6 \pm 0.6i$. This results in a frequency of $\omega _0 = 2.36$ rad/s. The magnitude of the poles will lead to slow exponential decay while the phase angle will induce intermediate sinusoidal behaviour as well as sign switching due to the negative component, resulting in a very high frequency.
**Case 3.**: The dominant poles are a set of complex conjugate poles with small positive real part, $z = 0.1 \pm 0.9i$. This results in a frequency of $\omega _0 = 1.46$ rad/s. The magnitude of the poles will lead to slow exponential decay while the phase angle will induce high frequency sinusoidal behaviour.
**Case 4.**: The dominant poles are a set of complex conjugate poles with positive real part, $z = 0.75 \pm 0.4i$. This results in a frequency of $\omega _0 = 0.49$ rad/s. The magnitude of most of the poles is close to one. This translates into a slower decay but larger amplitude changes.

**Figure 4:**
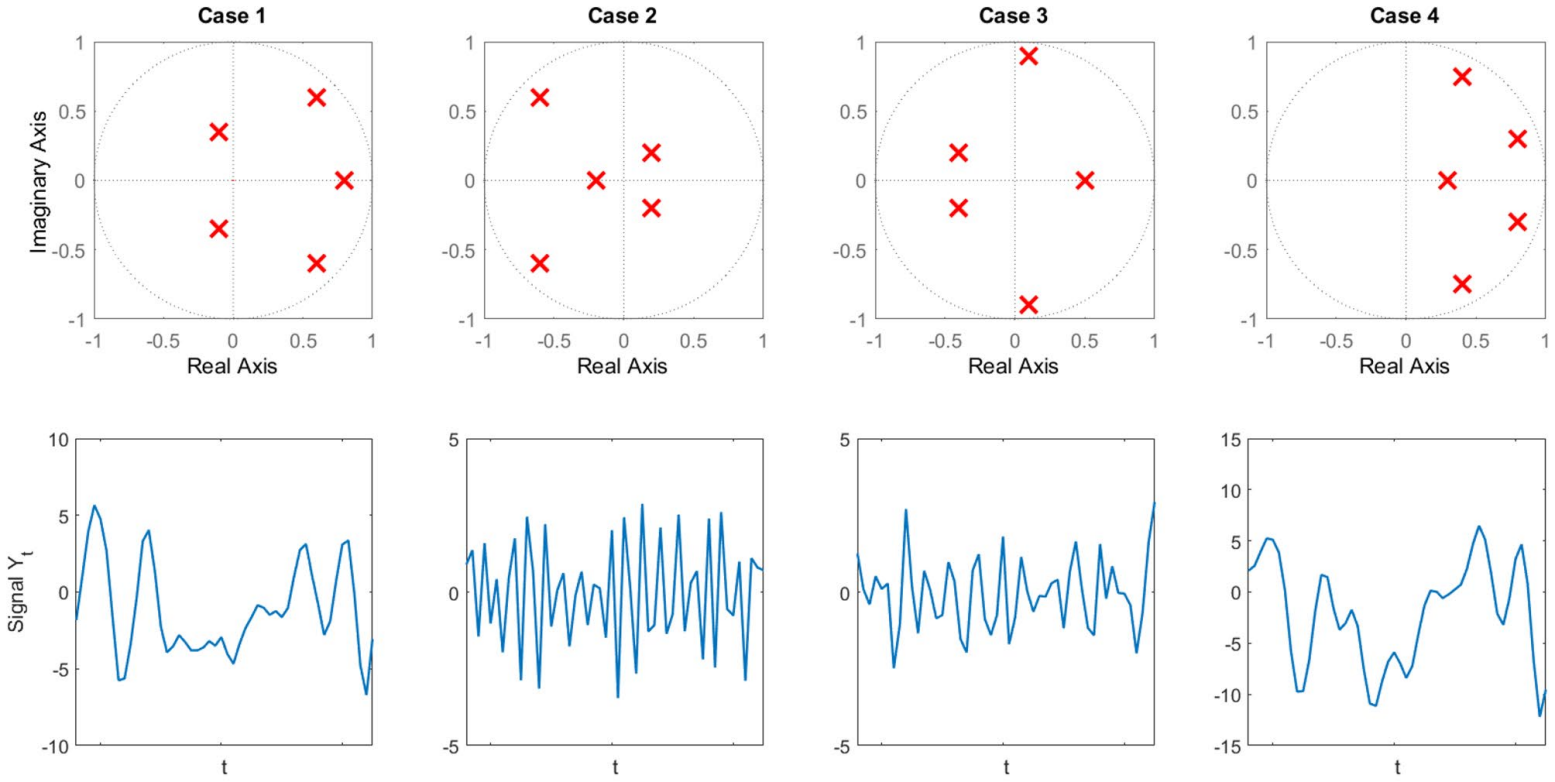
Pole-zero maps and observation samples for all cases. The first row contains the pole-zero maps of the four autoregressive processes. Poles are annotated with a $\times$. The second row contains a sample from a realization from each of the cases.

In Fig 4 the four cases are illustrated in a pole-zero map. Along with the pole-zero maps, for each case a time domain sample is displayed from a realization of the process generated using the mentioned poles.

Along with the benchmark criteria, we further consider the BVIC with two different sets of parameters to evaluate the penalization effect each term has on the forecasting performance. The sets are as follows: ($i$) $(\beta , \gamma ) = (1,1)$, ($ii$) $(\beta , \gamma ) = (5,1)$, and ($iii$) $(\beta , \gamma ) = (1,5)$. Finally, we conduct the Monte Carlo simulations for three different values of the noise parameter, $\delta$. Specifically, we chose the values of $\delta \in \{0, 0.1, 0.316\}$, which corresponds to a signal-to-noise ratio of SNR = $\infty$ dB (no noise), 10 dB, and 5 dB, respectively. In Table 1, we summarize the results from Experiment 1.

**Table 1:** Summary statistics for the Monte Carlo simulations conducted in Experiment 1.

	$\delta = 0$ (no noise)				$\delta = 0.1$ (10 dB)				$\delta = 0.316$ (5 dB)			
	MSE	VAR	${\overline{P}}_F$	${\overline{p}}$	MSE	VAR	${\overline{P}}_F$	$\bar{p}$	MSE	VAR	${\overline{P}}_F$	$\bar{p}$
**Case 1**												
$BVIC(\beta = 1, \gamma = 1)$	0.947	0.68	0.50	5.4	0.970	0.67	0.52	5.4	1.045	0.61	0.59	5.3
$BVIC(\beta = 5, \gamma = 1)$	0.941	0.67	0.55	4.1	0.958	0.66	0.56	4.1	1.021	0.59	0.63	4.0
$BVIC(\beta = 1, \gamma = 5)$	0.952	0.68	0.50	5.8	0.976	0.68	0.51	5.8	1.053	0.62	0.58	5.8
*AIC*	0.879	0.65	0.57	3.5	0.905	0.64	0.59	3.1	0.978	0.58	0.69	2.7
*BIC*	0.878	0.64	0.58	3.1	0.901	0.64	0.60	2.7	0.975	0.57	0.71	2.2
*AICc*	0.875	0.64	0.58	2.5	0.898	0.63	0.61	2.2	0.971	0.57	0.72	1.7
** Case 2**												
$BVIC(\beta = 1, \gamma = 1)$	0.848	0.49	0.67	5.3	0.855	0.49	0.68	5.3	0.898	0.48	0.70	5.3
$BVIC(\beta = 5, \gamma = 1)$	0.844	0.48	0.72	3.8	0.851	0.48	0.73	3.8	0.893	0.46	0.75	3.8
$BVIC(\beta = 1, \gamma = 5)$	0.852	0.50	0.66	5.8	0.859	0.50	0.67	5.8	0.901	0.48	0.70	5.8
*AIC*	0.812	0.46	0.69	3.1	0.822	0.46	0.70	3.1	0.876	0.45	0.74	3.0
*BIC*	0.811	0.46	0.70	2.6	0.822	0.46	0.71	2.6	0.876	0.45	0.76	2.5
*AICc*	0.808	0.45	0.71	2.1	0.819	0.46	0.72	2.1	0.873	0.45	0.77	2.0
**Case 3**												
$BVIC(\beta = 1, \gamma = 1)$	0.811	0.37	0.66	5.4	0.815	0.37	0.67	5.4	0.852	0.37	0.69	5.4
$BVIC(\beta = 5, \gamma = 1)$	0.822	0.37	0.72	3.9	0.825	0.37	0.72	3.9	0.858	0.37	0.74	3.9
$BVIC(\beta = 1, \gamma = 5)$	0.814	0.37	0.66	5.7	0.817	0.37	0.66	5.7	0.855	0.37	0.68	5.7
*AIC*	0.779	0.35	0.68	3.2	0.783	0.35	0.69	3.2	0.824	0.36	0.72	3.1
*BIC*	0.780	0.35	0.69	2.8	0.784	0.35	0.70	2.8	0.823	0.36	0.73	2.7
*AICc*	0.778	0.35	0.71	2.2	0.783	0.35	0.71	2.2	0.823	0.36	0.75	2.1
**Case 4**												
$BVIC(\beta = 1, \gamma = 1)$	0.749	0.52	0.52	5.5	0.779	0.54	0.54	5.4	0.869	0.52	0.60	5.3
$BVIC(\beta = 5, \gamma = 1)$	0.776	0.56	0.56	4.2	0.799	0.56	0.58	4.1	0.876	0.53	0.64	4.1
$BVIC(\beta = 1, \gamma = 5)$	0.742	0.51	0.52	5.9	0.771	0.53	0.53	5.8	0.865	0.51	0.59	5.8
*AIC*	0.740	0.54	0.55	4.7	0.788	0.57	0.58	3.6	0.887	0.55	0.69	2.8
*BIC*	0.743	0.54	0.55	4.4	0.793	0.57	0.59	3.1	0.890	0.55	0.71	2.2
*AICc*	0.751	0.55	0.56	3.8	0.802	0.58	0.61	2.4	0.894	0.55	0.73	1.7

The rows of the table display the criteria for each of the four cases. The columns show the metrics that we use to evaluate the criteria. Furthermore, the criteria are evaluated for different values of noise parameter $\delta$

#### Experiment 2

The objective is to assess the performance of the BVIC on a range of synthetic autoregressive time series of order $p \in \{10, 20, 30, 40, 50\}$. We conduct a Monte Carlo study in which we generate 100 windows with characteristics similar to those set in Experiment 1. Each window is generated by a set of poles that was generated randomly. For window $w_q$, with $q = 1, \dots , { n}_w$, complex conjugate poles are generated by randomizing a phase angle $\Phi _q$ and a magnitude $M_q$, where $0 \le \Phi _q \le \pi$, and $0.5 \le M_q < 1$. We define a set of complex conjugate poles with real and imaginary part described by $\alpha _q = M_q \cos \Phi _q$, and $\beta _q = M_q \sin \Phi _q$, respectively. To include the possibility of having real-valued poles, there is a 50 % chance that $\Phi _q$ is either 0 or $\pi$. For a detailed description on how the poles are generated, see Algorithm 1.

That said, regarding the remaining input parameters, we look at three different sets of hyperparameters for the BVIC: ($i$) $(\beta , \gamma ) = (1,1)$, and ($ii$) $(\beta , \gamma ) = (5,1)$. Simply speaking, the first puts equal weight on uncertainty, regression, forecasting and generalization, and the second more on forecasting and generalization. Furthermore, the noise parameter $\delta$ was set to 0.1 for this experiment.


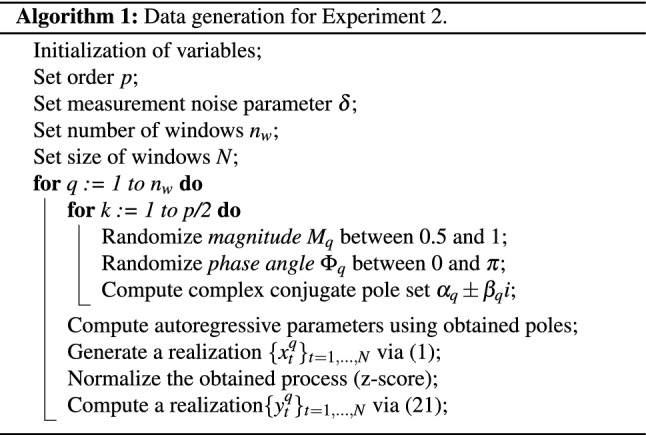


Assuming that autoregressive models of higher orders are also capable of forecasting over longer horizons, we initially evaluate the performance of each of the criteria over a forecast horizon $h_2 = p, \text { i.e., } h_2 \in \{10, 20, 30, 40, 50\}$ for each of the previously mentioned orders *p*, respectively. Additionally, to analyse the effects of the forecast horizon $h_2$, we conducted experiments where instead $h_2 = \text {ceil}(p/4), \text { i.e., } h_2 \in \{3, 5, 8, 10, 13\}$. Furthermore, to prevent a possible lack of observations for training of the BVIC, we increased the sample size to include more samples, thereby decreasing the prediction error. The amount of observations used to train models is derived functionally by $S_{{\mathcal {T}}} = 4.5(p + h_2)$. Thus, for $p=h_2$, the size of the training set becomes $S_{{\mathcal {T}}} = 9h_2$. A further split of $S_{{\mathcal {T}}}$ into training and validation for the BVIC gives a training, validation, and test ratio of 0.8, 0.1, and 0.1, respectively. The results of Experiment 2 are summarized in Table 2.

**Figure 5:**
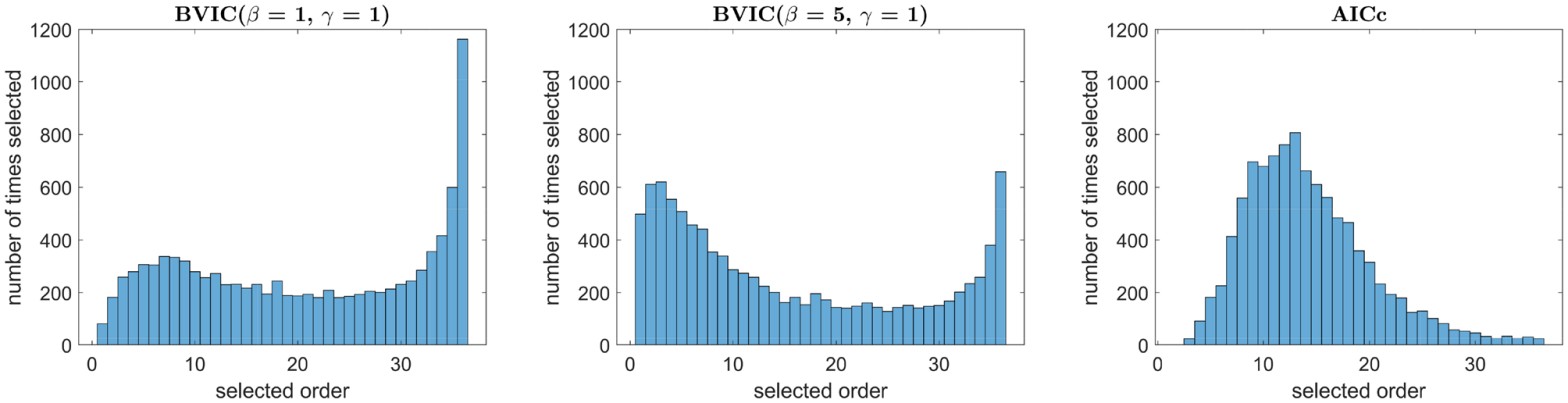
Distribution of orders. This figure depicts the distribution of orders selected by the BVIC with $\beta = 1$, the BVIC with $\beta = 5$, and AICc for the case where $p = 30$ in Experiment 2.

Moreover, to give a clearer image of what orders are being selected by the criteria, a graphical representation of the distributions is showed in Fig 5. Here, we have plotted the histograms of all the orders that were selected in all simulations by the BVIC with two different sets of hyperparameters, and AICc.

**Table 2:** Summary statistics for the Monte Carlo simulations conducted in Experiment 2.

	$h_2 = p$				$h_2=\text {ceil}(p/4)$			
	MSE	VAR	${\overline{P}}_F$	${\overline{p}}$	MSE	VAR	${\overline{P}}_F$	${\overline{p}}$
**AR(10)** $(p=10)$								
$BVIC (\beta = 1, \gamma = 1)$	0.388	0.28	0.34	8.0	0.153	0.09	0.16	7.5
$BVIC (\beta = 5, \gamma = 1)$	0.392	0.28	0.36	6.2	0.161	0.10	0.17	6.0
*AIC*	0.373	0.26	0.34	7.0	0.150	0.09	0.16	7.2
*BIC*	0.373	0.26	0.35	4.0	0.147	0.08	0.16	4.1
*AICc*	0.372	0.26	0.34	6.2	0.148	0.09	0.16	6.3
**AR(20)** $(p=20)$								
$BVIC (\beta = 1, \gamma = 1)$	0.352	0.27	0.30	14.4	0.093	0.04	0.10	13.5
$BVIC (\beta = 5, \gamma = 1)$	0.353	0.27	0.31	11.0	0.096	0.04	0.11	10.3
*AIC*	0.336	0.25	0.30	12.7	0.090	0.03	0.10	12.7
*BIC*	0.338	0.25	0.31	5.4	0.091	0.03	0.11	5.7
*AICc*	0.334	0.25	0.30	10.9	0.089	0.03	0.10	10.9
**AR(30)** $(p=30)$								
$BVIC (\beta = 1, \gamma = 1)$	0.375	0.28	0.33	20.7	0.085	0.03	0.10	19.2
$BVIC (\beta = 5, \gamma = 1)$	0.377	0.28	0.34	15.5	0.089	0.04	0.10	14.5
*AIC*	0.358	0.26	0.33	16.8	0.083	0.03	0.10	17.0
*BIC*	0.359	0.25	0.34	7.1	0.083	0.03	0.10	6.8
*AICc*	0.356	0.25	0.33	14.3	0.082	0.03	0.10	14.4
** AR(40)** $(p=40)$								
$BVIC (\beta = 1, \gamma = 1)$	0.381	0.27	0.33	27.5	0.061	0.01	0.08	24.4
$BVIC (\beta = 5, \gamma = 1)$	0.385	0.28	0.35	20.3	0.063	0.01	0.08	17.8
*AIC*	0.364	0.26	0.33	21.0	0.058	0.01	0.07	20.3
*BIC*	0.371	0.26	0.35	8.2	0.060	0.01	0.08	8.1
*AICc*	0.363	0.25	0.34	17.8	0.057	0.01	0.07	17.1
**AR(50)** $(p=50)$								
$BVIC (\beta = 1, \gamma = 1)$	0.362	0.26	0.31	32.1	0.058	0.01	0.07	29.6
$BVIC (\beta = 5, \gamma = 1)$	0.368	0.27	0.33	23.4	0.061	0.01	0.08	21.5
*AIC*	0.339	0.24	0.31	23.5	0.055	0.01	0.07	22.7
*BIC*	0.351	0.24	0.33	8.9	0.057	0.01	0.08	9.2
*AICc*	0.339	0.24	0.31	20.1	0.054	0.01	0.07	19.3

This table contains summary statistics for each information criterion for higher model orders and larger forecast horizons. We conducted one-way analysis of variance (ANOVA) tests and Kruskal-Wallis (KW) tests to assess if the error distributions found in the experiments were statistically distinguishable. Specifically, we could not find any statistically significant difference based on the one-way ANOVA and KW tests with a 0.05 significance level. These findings are further detailed in S3 Supplementary Information

#### Experiment 3

Hereafter, we aim to test the predictability of ECoG data during epileptic events. As such, we analyse the ability of the previously discussed criteria to forecast sections of data corresponding to seizures and non-seizures.

Similarly to the previous experiments, sections are extracted from the time series that are subsequently split up into windows. These windows have a total of $N = 1000$ samples such that we can effectively use 800 (80$\%$) samples for training, 100 (10%) for validation, and 100 (10$\%$) samples for testing. The choice of *N* comes from a sensitivity analysis that we present in detail in S4 Supplementary Information. Next, we perform statistical tests to assess the stationarity of the ECoG recordings used in this experiment. We found that a sample size of $N=1000$ is a suitable amount that results in sufficient evidence for stationarity in the majority of the considered windows.

Differences in scaling are found in recordings from different patients and between ictal and interictal phases of a single patient. Therefore, to facilitate a fair comparison among the different data, we use the mean absolute scaling error (MASE)^17^ as a metric to compare the results from this experiment. The advantage of using the MASE over metrics such as MSE is that the prior is scale-independent. The mean absolute scaled error is formulated as

26 $$\begin{aligned} \mathrm {MASE} = \frac{\frac{1}{h_2-h_1+1}\sum _{t=n+h_1}^{n+h_2}|Y_t-{\hat{Y}}_t|}{\frac{1}{n-1}\sum _{t=2}^n |Y_{t} - Y_{t-1}|}. \end{aligned}$$

Simply speaking, the MASE is constructed by dividing the mean absolute error (MAE) by the average naïve forecast computed in-sample. Thus, for a single forecast horizon, a MASE of less than one implies that the forecast is better than the average in-sample naïve forecast. Furthermore, we establish that the performance of the BVIC in comparison to other information criteria is similar, without any statistically significant difference in the majority of the simulations—see details in S3 Supplementary Information. Therefore, for this experiment we consider only the BVIC with $\beta = 1$, and $\gamma = 1$, to assess the ability of the BVIC to forecast electrocorticography data.

**Figure 6:**
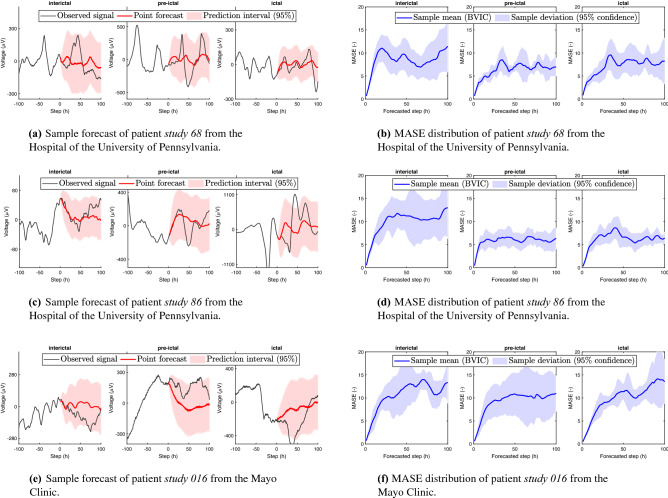
Interictal, pre-ictal, and ictal forecast error. Comparison of mean absolute scaled error (MASE) obtained by the BVIC for 1- to 100-step ahead forecasts. The solid blue line indicates the mean MASE across all channels. The blue shaded areas indicate the range containing the deviations along the considered channels, with a 95% certainty. Additionally, sample forecasts with point forecast and prediction intervals are depicted in (**a**), (**c**), and (**e**). Note that the forecast step $h_2$ is indicated with *h* in the figure.

The results of the experiment are plotted in Fig. 6. Here, we plotted the average MASE over the channels in which a seizure was identified for single forecast horizons ranging from 1 to 100 steps into the future. Figure 6a,c,e contain a sample forecast with red color, while Fig.  6b,d,f depict in blue the average MASE with the variance among different channels. Therefore, it is worth noticing that the red shading in Fig 6a,c,e indicates a prediction interval, and shows the estimated interval in which the forecasted observation is within 95% certainty. Whereas, the blue shading in Fig.  6b,d,f is simply showing the interval in which 95% of the computed values are contained (i.e., $\pm 1.96\sigma$).

## Discussion

We introduced a principled analysis of an information criterion that utilizes theoretical principles of time-reversibility and time series to assemble a finite-sample data-driven approach to model selection that eliminates the penalization of the model order and replaces it with a backward validation scheme that can be tuned to trade-off between uncertainty, regression, generalization and forecasting.

### Information criteria performance

Experiment 1 explores pedagogical examples to capture the behavior of the different information criteria when different pole locations and signal-to-noise ratios of the time series are considered. It is possible to notice that these impact the performance of the BVIC relative to the other information criteria. For instance, from *Case 1* we notice that for systems with a large real-valued pole ($z_i = 0.9$), the BVIC predicts with larger error compared to the other criteria. On the other hand, when all poles have large absolute values ($|z| < 0.6$), such as in *Case 4*, we observe a relative decrease in prediction error of the BVIC, especially when the signal-to-noise ratio is larger than zero.

Moreover, we find that the BVIC selects larger orders than the other criteria, on average. Here, we noticed is that the BVIC shows a certain consistency over all the simulations. For $\beta = 1$, the BVIC finds approximately the true order of 5. Whereas for $\beta = 5$, the average order selected is roughly 3.6. On the contrary, the AIC, BIC, and AICc all have much larger variance in their average selected orders. Thus, in contrast with the three other criteria, the BVIC is more consistent in selecting the order, independent of the location of the poles and the variance of the measurement noise.

In Experiment 2, we provide converging evidence that the different information criteria perform in a similar fashion to the BVIC. Nonetheless, it is important to emphasize that the BVIC provides a principled method that relies on finite samples and offers a trade-off between uncertainty, regression, generalization and forecasting, in contrast with other information criteria where the penalization term is fixed to satisfy asymptotic properties^10^. Specifically, based on statistical tests, namely the one-way analysis of variance and the Kruskal-Wallis test, we found that none of the obtained distributions were significantly different between the criteria at a significance level of 0.05.

### Model order selection in autoregressive models with the BVIC

It is interesting to notice how the BVIC is able to capture a different range of orders across the different synthetically generated data—see Fig 5. Remarkably, we also notice that the BVIC selects orders that are, on average, closer to the true order of the synthetic process—see Tables 1 and 2. Thus, providing evidence that the BVIC may be a preferable method when it comes to estimating the true order of an autoregressive model. Lastly, the BVIC is also able to adapt to a changing forecast horizon, where a shorter forecast horizon means that the BVIC selects, on average, lower orders. On the other hand, the selection of the orders by the remaining information criteria is not influenced by the forecast horizon.

Given that the BVIC selects model orders that are, on average, closer to the true order of the model, in Experiment 3, we tested the ability of the BVIC to assess the *memory order* (i.e., the statistical significant dependency or previous realizations of the time series) in the context of seizure prediction^6^.

Furthermore, there has been long reported evidence that the memory order increases during the ictal state^18,19^. Implicitly, an increase in memory would also indicate an increase in the number of steps for which we can forecast ahead. In Experiment 3, we collected converging evidence towards the later points, as the predictability increases during the pre-ictal and ictal state compared with the interictal state.

Nonetheless, it is worth reporting that this is not always the case, as can be seen in Fig. 6f, where there is no significant decrease in error between the different states. The reason for the irregularity is something that would require more study. However, there are a few possible causes that may attribute to this outcome. First of all, we provided evidence for the stationarity of the three recordings that were used in the experiment—see S4 Supplementary Information. Nevertheless, the recording from patient study 016 showed the weakest evidence for stationarity. Thus, certain amount of non-stationarity in the data could ascribe to the differing results seen in Fig. 6f. Secondly, following up from the previous argument, we must consider that it might not be possible to predict seizures with a single framework due to the variety of mechanisms that underlie an epileptic seizure. Finally, considering the fact that the average MASE obtained for study 016 is similar for all states, it could be that the electrodes that were identified as seizure electrodes were not actually placed in the location of the seizure, or, the entire recording was incorrectly classified as a seizure^20^.

### Extensions and future work

Whereas we have focused on the univariate autoregressive models, it would be interesting to extend the BVIC to a multivariate setting. This may reveal to be beneficial when the underlying dynamics captured by a multivariate time series have spacial dependencies. For instance, this is the case of ECoG recordings explored in Experiment 3, where there is evidence that a seizure propagates through the brain and reveals itself across different channels over time. Additionally, it would be worth to expand the proposed information criteria to handle both moving average and fractional integrative models known to be able to handle long-term memory^13^. Furthermore, the BVIC is a data-driven model, therefore, it can be used in different contexts. For instance, it would be interesting to find an equivalent to the BVIC to determine the minimum number of parameters for deep learning models that are capable to guarantee forecasting capabilities. Lastly, it would be interesting to see if it would be possible to have a recursive method for online implementation such that it becomes dynamic in nature.

## Supplementary Information

Supplementary Information 1.

Supplementary Information 2.

Supplementary Information 3.

Supplementary Information 4.

Supplementary Information 5.

## Data Availability

All the data and algorithms used to produce the plots in the manuscript are available at https://github.com/maxsibeijn/BVIC_MatlabFiles. All patient data was obtained from the IEEG Portal (IEEG.ORG). The IEEG Portal is a collaborative initiative between the National Institutes of Neurological Disorders and Stroke, Mayo Clinic and the Hospital of the University of Pennsylvania. All data that was used is publicly available. These organizations handle strict guidelines and regulations for their methods. Their experimental protocols are approved, and informed consent is required from all subjects.
