# Supplementary material for: A time-reversed model selection approach to time series forecasting
Source: Sci Rep. 2022 Jun 28;12:10912. doi: 10.1038/s41598-022-15120-x (PMC9240029; doi:10.1038/s41598-022-15120-x)
Supplement: Supplementary file 1 — Supplementary Information 1. [file 41598_2022_15120_MOESM1_ESM.pdf]

S1 Supplementary Information
“A time-reversed model selection approach to time series forecasting”

Max Sibeijn[1,*] **and Sérgio Pequito**[1]

[1]Delft Center for Systems and Control, Delft University of Technology, Delft, The Netherlands
[*]m.w.sibeijn@tudelft.nl

S1. The BVIC derivation

The BVIC consists in the integration and unification of four components. The first component is the *log-likelihood function*. The log-likelihood function can be used to compute the MLE. The MLE is the set of parameters for which the log-likelihood is maximized. For an autoregressive model $j \in \mathscr{M}$ with parameters $\theta_j \in \mathbb{R}^p$, where $p \in \mathbb{N}$, the log-likelihood may be expressed as[2]

$$\ell(\theta_j) = -\frac{n}{2}\log \hat{\sigma}(\theta_j)^2, \tag{1}$$

where $n \in \mathbb{N}$ is the number of observations and $\hat{\sigma}(\theta_j)^2 = \frac{1}{n}\sum_{i=1}^{n}(X_i - X_i^j)^2$ is the average ℓ_2-loss function for all observations.

Accordingly, an additional component is required that prevents over-fitting inherent to the MLE. Using the results from the section on the reversibility of time series, where we show that the backwards linear prediction may be used as a proxy for the forward linear prediction, we propose to utilize the backwards prediction to assess the forecast capabilities of candidate models. We consider two metrics to quantify the performance of the backwards prediction: (*i*) the backcast error, and (*ii*) the backcast uncertainty. More specifically, we consider the mean square backcast error (MSBE) and the backcast variance as suitable metrics for evaluation.

Consider an arbitrary autoregressive process where we want to apply the previously described metrics. First, the mean square backcast error may be obtained by taking the mean of the squared error over backcast horizon (h_1, h_2), i.e., $\mathrm{err}(\theta_j) = \frac{1}{h_2 - h_1 + 1}\sum_{t=h_1}^{h_2} e_{1-t}^2$, where e_{1-t} is depicted in the green dotted lines in Fig S1a. Second, the backcast variance represents the variance associated with the backwards prediction for each step. Specifically, we use the average variance over the backcast horizon, illustrated in Fig S1b with red dotted lines, which can be described functionally as $\mathrm{var}(\theta_j) = \frac{1}{h_2 - h_1 + 1}\sum_{t=h_1}^{h_2} P_{1-t}^n$.

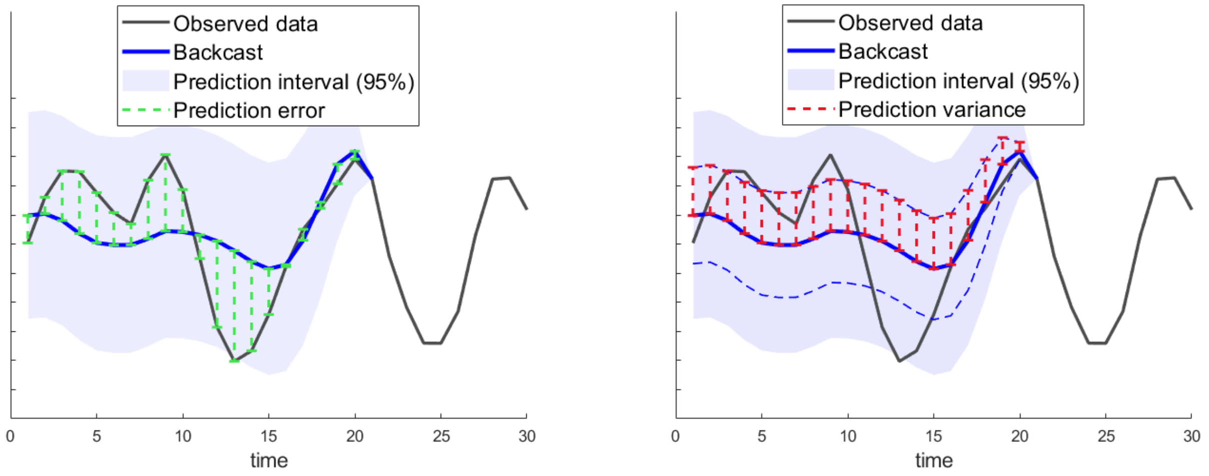

(a) Sample backcast with the error at each time step indicated with a dotted red line.

(b) Sample backcast with the prediction variance at each time step indicated with a magenta line.

S1 Fig. Backcasting metrics. An illustration of the metrics that we use to quantify the backcasting performance of a specific model.

The last component we consider for the novel criterion is the *model order*. To counteract the risk of over-fitting, classical criteria incorporate the model order p as a penalty variable in their model selection procedure. Consequently, over-fitting is reduced by penalizing higher orders, resulting in increased parsimony of selected models. Arguably, the manner in which model complexity is penalized may differ depending on the application. However, classical information criteria operate with a static penalty term, therefore, they are susceptible irregularities and differences between datasets. This results in the absence of a uniformly preferable criterion[3], and leaves it up to the user to determine the best criterion for their data. Considering the data-driven approach to obtaining generalization capabilities that was introduced in the former paragraphs, we decided to exclude the order penalization component from the novel criterion.

Altogether, excluding the model order, we are left with three components. From these three components, a weighted combination is constructed that represents the basic structure of the novel criterion, as follows:

$$-a \times \frac{\ell(\theta_j)}{n} + b \times \mathrm{err}(\theta_j) + c \times \mathrm{var}(\theta_j), \tag{2}$$

where a, b, and c are real-valued positive scalars. Technically, θ_j depends on the horizon of the backcast h, therefore, the backcasting components should be $\mathrm{err}(\theta_j^{(1)}, \ldots, \theta_j^{(h)})$ and $\mathrm{var}(\theta_j^{(1)}, \ldots, \theta_j^{(h)})$. However, for simplicity we only use θ_j to indicate the index variable.

The expression in (2) has three parameters. To simplify the multi-objective problem we reduce the number of parameters and normalize the expression. Firstly, consider a simplification step to remove a parameter, a, from the equation in (2). For the purpose of model selection, we are only interested in the relative differences between the terms in the criterion. Therefore, it is possible to set α as a constant, while keeping the other two variable. Secondly, each of the terms in the equation is normalized by dividing each expression with a baseline constant value. This value is computed by estimating the parameters θ_m with a fixed order $m = \max_{\{j \in \mathscr{M}\}} j$. Hence, we obtain

$$\begin{aligned} a &= \frac{n}{|\ell(\theta_m)|}, \\ b &= \frac{\beta}{\mathrm{err}(\theta_m)}, \text{ and} \\ c &= \frac{\gamma}{\mathrm{var}(\theta_m)}. \end{aligned} \tag{3}$$

Depending on the scale of the data in the time series, the magnitude of the MLE might be positive or negative. Therefore, to ensure that the normalization does not flip the sign of the objective function, the absolute value of $\ell(\theta_m)$ is used.

Finally, the results from (3) can be substituted into the expression in (2) to obtain a final criterion. The BVIC is given by

$$\mathrm{BVIC}(\theta_j, j) = -\frac{\ell(\theta_j)}{|\ell(\theta_m)|} + \beta \frac{\mathrm{err}(\theta_j)}{\mathrm{err}(\theta_m)} + \gamma \frac{\mathrm{var}(\theta_j)}{\mathrm{var}(\theta_m)}, \tag{4}$$

where $\beta, \gamma \geq 0$ are tunable hyperparameters that can be used to increase the importance of each of the three terms in the expression.

Parameter Estimation. Now that the structure of the BVIC has been decided upon, a method for estimating the parameters must be chosen. we split the estimation of the parameters up into two parts and compute the parameters through separate estimation techniques, detailed next. Hence, the BVIC becomes an index criterion instead of a multi-objective optimization criterion, similar to AIC and BIC. Specifically, an estimate of the indices is computed for different model orders and the one that attains a minimum is considered.

Therefore, for the log-likelihood term we use parameters estimated through MLE to obtain a measure of goodness-of-fit[2]. Here, the argument of (1) is maximized as follows:

$$\hat{\theta}_j = \arg\max_{\theta_j} \ell(\theta_j), \tag{5}$$

where $\hat{\theta}_j \in \mathbb{R}^p$ is the set of parameters for which the log-likelihood function is maximized, i.e., the MLE. As such, the function $\ell(\hat{\theta}_j)$ is essentially a measure of *goodness-of-fit* of the model parameters upon the observed data.

Secondly, as is common in forecasting, we estimate the parameters for the backcasting error and variance using the Yule-Walker equations[1]. The Yule-Walker equations were discussed previously in the section on reversibility of time series. This technique is advantageous because it can compute both point estimates and prediction variance. Additionally, this method can be used to estimate parameters for multi-step forecasts. The parameters obtained with Yule-Walker are found by

$$\hat{\theta}_j^{yw} = \Gamma_j^{-1}\gamma_j, \tag{6}$$

where $\Gamma_j \in \mathbb{R}^{j\times j}$ is the autocovariance matrix, and $\gamma_j \in \mathbb{R}^j$ is the vector of autocovariances containing j lags.

Altogether, the estimated parameters can be used to formulate a mathematical objective criterion to determine the order of an autoregressive model. This criterion is

$$\mathrm{BVIC}(j) = -\frac{\ell(\hat{\theta}_j)}{|\ell(\hat{\theta}_m)|} + \beta\frac{\mathrm{err}(\hat{\theta}_j^{yw})}{\mathrm{err}(\hat{\theta}_m^{yw})} + \gamma\frac{\mathrm{var}(\hat{\theta}_j^{yw})}{\mathrm{var}(\hat{\theta}_m^{yw})}, \tag{7}$$

where $\beta, \gamma \geq 0$. Thus, the model order is obtained through minimization of the BVIC, as follows:

$$j^* = \arg\min_{j\in\mathscr{M}} \mathrm{BVIC}(j). \tag{8}$$

References

1. Box GE, Jenkins GM, Reinsel GC, and Ljung GM. Time series analysis: forecasting and control. John Wiley & Sons, 2015.
2. Konishi S, Kitagawa G. Information criteria and statistical modeling. Springer Science & Business Media, 2008.
3. McQuarrie AD, Tsai CL. Regression and time series model selection. World Scientific, 1998.
