# Supplementary material for: A time-reversed model selection approach to time series forecasting
Source: Sci Rep. 2022 Jun 28;12:10912. doi: 10.1038/s41598-022-15120-x (PMC9240029; doi:10.1038/s41598-022-15120-x)
Supplement: Supplementary file 2 — Supplementary Information 2. [file 41598_2022_15120_MOESM2_ESM.pdf]

S2 Supplementary Information
"A time-reversed model selection approach to time series forecasting"

Max Sibeijn[1,*] **and Sérgio Pequito**[1]

[1]Delft Center for Systems and Control, Delft University of Technology, Delft, The Netherlands
[*]m.w.sibeijn@tudelft.nl

S2. Least-squares estimation

Let us pose the autoregressive process of order p as a dynamical system, consider the state space form

$$x_{k+1} = Ax_k + \varepsilon_k, \tag{1}$$

where $x_k, \varepsilon_k \in \mathbb{R}^p$ are vectors containing lagged values of the state and noise, respectively. The system matrix is denoted by $A \in \mathbb{R}^{p \times p}$. Writing out the equation into matrix form results in

$$\begin{bmatrix} x(k+1) \\ x(k) \\ \vdots \\ x(k-p+1) \end{bmatrix} = \underbrace{\begin{bmatrix} -a_1 & -a_2 & -a_3 & \dots & -a_p \\ 1 & 0 & 0 & \dots & 0 \\ 0 & 1 & 0 & \dots & 0 \\ \vdots & \vdots & \ddots & \ddots & \vdots \\ 0 & 0 & \dots & 1 & 0 \end{bmatrix}}_{A} \begin{bmatrix} x(k) \\ x(k-1) \\ \vdots \\ x(k-p) \end{bmatrix} + \begin{bmatrix} \varepsilon(k+1) \\ \varepsilon(k) \\ \vdots \\ \varepsilon(k-p+1) \end{bmatrix}. \tag{2}$$

Note that the A matrix is written in companion form, resulting in the autoregressive parameters to be contained in the top row of the matrix.

To find the A matrix a least-squares problem is formulated that minimizes ε_k as follows:

$$\min ||\varepsilon_k||^2 = \min_A ||x_{k+1} - Ax_k||^2 \tag{3}$$

To better capture the dynamical behaviour it is best to increase the amount of data used for the least squares estimator. Therefore, variables x_{k+1} and x_k are used to construct Hankel matrices $H_{x,k+1}$ and $H_{x,k}$. The least squares problem becomes

$$|| \underbrace{\begin{bmatrix} x(k+1) & x(k) & \dots & x(p+1) \\ x(k) & x(k-1) & \dots & x(p) \\ \vdots & \vdots & \ddots & \vdots \\ x(k-p+1) & x(k-p) & \dots & x(1) \end{bmatrix}}_{H_{x,k+1}} - A \underbrace{\begin{bmatrix} x(k) & x(k-1) & \dots & x(p) \\ x(k-1) & x(k-2) & \dots & x(p-1) \\ \vdots & \vdots & \ddots & \vdots \\ x(k-p) & x(k-p-1) & \dots & x(0) \end{bmatrix}}_{H_{x,k}} ||_F^2. \tag{4}$$

The solution of the least squares problem can be denoted as

$$\hat{A} = H_{x,k+1} H_{x,k}^\top (H_{x,k} H_{x,k}^\top)^{-1}. \tag{5}$$
