# Supplementary material for: A time-reversed model selection approach to time series forecasting
Source: Sci Rep. 2022 Jun 28;12:10912. doi: 10.1038/s41598-022-15120-x (PMC9240029; doi:10.1038/s41598-022-15120-x)
Supplement: Supplementary file 3 — Supplementary Information 3. [file 41598_2022_15120_MOESM3_ESM.pdf]

S3 Supplementary Information
“A time-reversed model selection approach to time series forecasting”

Max Sibeijn[1,*] and Sérgio Pequito[1]

[1]Delft Center for Systems and Control, Delft University of Technology, Delft, The Netherlands
[*]m.w.sibeijn@tudelft.nl

S3. Statistical tests for difference among samples

We perform two tests to evaluate whether there is a statistically significant difference in the error distributions obtained in Experiment 2. Specifically, we compare the single step forecast error distributions resulting from the information criteria in the AR(30) process. The tests performed are as follows:

> **One-way analysis of variance (ANOVA) test.** Assesses the null hypthesis that the samples of a set of groups (i.e., information criteria) are drawn from populations with the same mean.
>
> **Kruskal-Wallis (KW) test.** Assesses the null hypothesis the samples of a set of groups (i.e., information criteria) are drawn from the distributions with the same median.

In Fig S3a and Fig S3b respectively, we have plotted the results of the ANOVA test and the KW test into a box-plot that depicts the distribution of p-values for 100 Monte Carlo simulations, for forecast horizons ranging from 1 to 30. Here, for both ANOVA and KW, the p-values do not drop below the 0.05 significance for any of the forecast horizons. Thus, there is not enough statistical evidence in support of rejecting the null hypotheses.

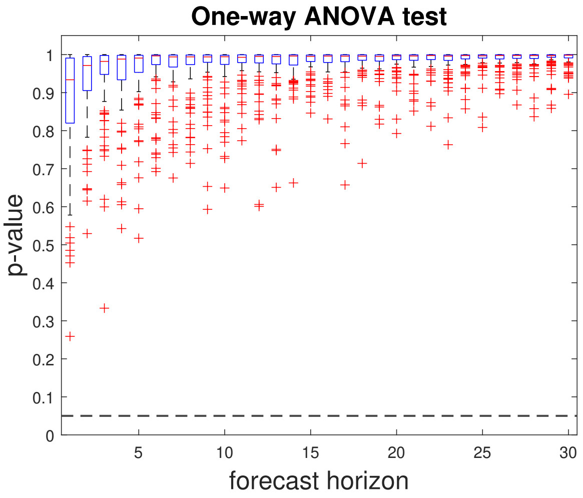

(a) Box-plot of p-value distribution when performing One-way ANOVA.

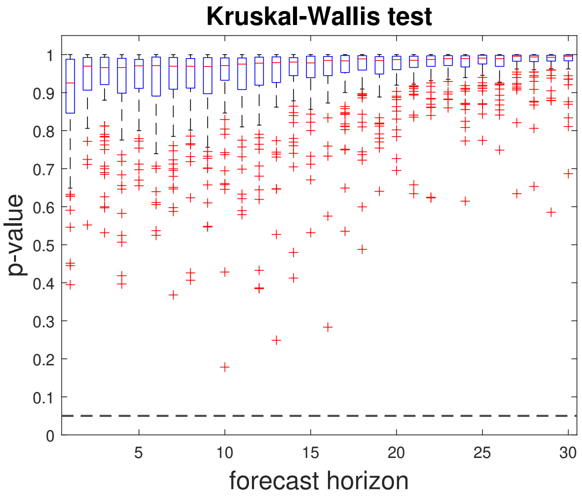

(b) Box-plot of p-value distribution when performing Kruskal-Wallis.

S3 Fig. Statistical tests. Box-plots depicting the spread of p-values obtained by conducting one-way ANOVA and Kruskal-Wallis tests on 100 distributions of mean square forecast error for all single forecasts horizons. The 0.05 significance level is indicated with a dotted black line.
