# Supplementary material for: A time-reversed model selection approach to time series forecasting
Source: Sci Rep. 2022 Jun 28;12:10912. doi: 10.1038/s41598-022-15120-x (PMC9240029; doi:10.1038/s41598-022-15120-x)
Supplement: Supplementary file 4 — Supplementary Information 4. [file 41598_2022_15120_MOESM4_ESM.pdf]

# S4 Supplementary Information
# "A time-reversed model selection approach to time series forecasting"

**Max Sibeijn**[1,*] **and Sérgio Pequito**[1]

[1]Delft Center for Systems and Control, Delft University of Technology, Delft, The Netherlands
[*]m.w.sibeijn@tudelft.nl

## S4. Statistical tests for stationarity

An implicit assumption of the BVIC (and all other criteria) is stationarity of the time series. That said, ECoG recordings are said to be approximately stationary over limited periods of time[1]. In this appendix we conduct statistical test for stationarity to determine what would be a favorable window size. Two hypothesis tests are conducted, where one tests for stationarity while the other tests for non-stationarity. Specifically, the tests conducted are as follows:

**Leybourne-McCabe (LMC) test.** Assesses the null hypthesis that a time series is a trend stationary AR($p$) process. $H_0$: $Y_j$ is stationary.

**Phillips-Perron (PP) test.** Assesses the null hypothesis that a unit root is present in a time series. $H_0$: $Y_j$ is non-stationary.

Ultimately, a failure to reject the null hypothesis of the LMC test and a rejection of the PP test null hypothesis at a 0.05 significance level would be enough evidence to suggest that the time series is stationary. Therefore, we conduct the previously mentioned statistical test on the three different ECoG recordings, where each recording is divided into windows of length $N$. Over all the windows, we evaluate the percentage of time in which the null hypothesis is rejected, i.e., $r_{H_0} = \frac{\#\text{times } H_0 \text{ is rejected}}{\text{total windows}}$. The results are shown in Fig S4. Noticeably, the ratio of rejection converges to the preferred outcome. However, it seems that for the patient study 16 from the Mayo Clinic we see less evidence to reject non-stationarity, and more evidence to reject stationarity.

In summary, we may be inclined to choose $N$ as large as possible. However, we are limited to the length of sections of similar behaviour that we are able to use. This is due to shifting states of the brain, from interictal to pre-ictal, and from the latter to ictal. Commonly, the duration of the pre-ictal state constitutes of around 10 to 20 thousands samples. Consequently, we would like to limit the size of the windows such that we can still have enough windows to perform simulations on. Therefore, we have chosen a window size of $N = 1000$ to be suitable for the data that we are considering, even though there is some evidence for non-stationarity in one of the recordings.

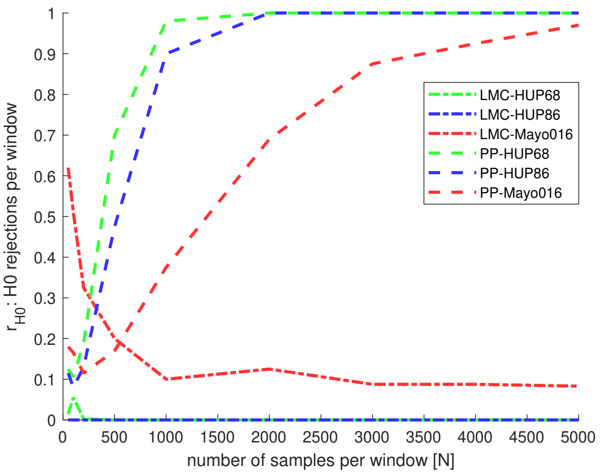


**S4 Fig. Stationarity tests.** Leybournce-McCabe and Phillips-Perron test outcomes represented as the fraction of rejections over the total amount of tests performed for each of the ECoG recordings evaluated in Experiment 3.

## References


1. Murin Y, Goldsmith A, Aazhang B. Estimating the memory order of electrocorticography recordings. IEEE Transactions on Biomedical Engineering, 2019. 66(10), 2809-2822.
2. Shumway RH, Stoffer DS. ARIMA models. In Time series analysis and its applications. Springer, 2017. 75-163.
