# Supplementary material for: A time-reversed model selection approach to time series forecasting
Source: Sci Rep. 2022 Jun 28;12:10912. doi: 10.1038/s41598-022-15120-x (PMC9240029; doi:10.1038/s41598-022-15120-x)
Supplement: Supplementary file 5 — Supplementary Information 5. [file 41598_2022_15120_MOESM5_ESM.pdf]

# S5 Supplementary Information
# “A time-reversed model selection approach to time series forecasting”

**Max Sibeijn**[1,*] **and Sérgio Pequito**[1]

[1]Delft Center for Systems and Control, Delft University of Technology, Delft, The Netherlands
[*]m.w.sibeijn@tudelft.nl

## S5. Theorem 1

(Shumway et al.[1]) *Let U,V be discrete random variables. For any function* $g : \mathbb{R} \to \mathbb{R}$,

$$E\left[(V - E[V \mid U])^2\right] \leq E\left[(V - g(U))^2\right],$$

*and we have equality if and only if* $g(U) = E[V \mid U]$.

## References

1. Shumway RH, Stoffer DS. ARIMA models. In Time series analysis and its applications. Springer, 2017. 75-163.
